# Molecular adaptation and expression evolution following duplication of genes for organellar ribosomal protein S13 in rosids

**DOI:** 10.1186/1471-2148-8-25

**Published:** 2008-01-26

**Authors:** Shao-Lun Liu, Keith Adams

**Affiliations:** 1 UBC Botanical Garden & Centre for Plant Research and Botany Department, University of British Columbia, Vancouver, BC, V6T1Z4, Canada

## Abstract

**Background:**

Gene duplication has been a fundamental process in the evolution of eukaryotic genomes. After duplication one copy (or both) can undergo divergence in sequence, expression pattern, and function. Two divergent copies of the ribosomal protein S13 gene (*rps13*) of chloroplast origin are found in the nucleus of the rosids *Arabidopsis*, *Gossypium*, and *Glycine*. One encodes chloroplast-imported RPS13 (nucp *rps13*), while the other encodes mitochondria-imported RPS13 (numit *rps13*). The *rps13 *gene has been lost from mitochondrial DNA (mt *rps13*) of many rosids.

**Results:**

We studied sequence evolution of numit *rps13 *in comparison with nucp *rps13 *in seven rosid genera. K_a_/K_s _analysis and likelihood ratio tests showed considerably higher K_a _values and K_a_/K_s _ratios in numit *rps13 *than in nucp *rps13*, indicating increased amino acid sequence divergence in numit *rps13*. Two positively selected codons were detected in numit RPS13 in regions that are inferred to interact with the 16S rRNA. Several amino acids in numit RPS13 have changed from the one present in nucp RPS13 to the one present in mt RPS13, showing that numit *rps13 *is becoming more like mt *rps13*. Comparison of expression patterns and levels of numit *rps13 *and nucp *rps13 *in *Arabidopsis *using microarray data indicated divergence in gene expression. We discovered that in addition to numit *rps13*, *Malus *(apple) contains a transcribed mt *rps13 *gene. To determine if partitioning of expression takes place between numit *rps13 *and mt *rps13*, expression of both copies and RNA editing of mt *rps13 *were examined by RT-PCR, qRT-PCR, and sequencing from 14 different organ types plus seedlings subjected to five different abiotic stresses. Co-expression of numit *rps13 *and mt *rps13 *was observed in all the organs and various stress treatments. We determined that purifying selection is acting on both numit *rps13 *and mt *rps13 *in *Malus*.

**Conclusion:**

Our data provide evidence that numit *rps13 *genes in rosids have experienced adaptive sequence evolution and convergent evolution with mt *rps13*. Co-expression of numit *rps13 *and mt *rps13 *and purifying selection on both genes in *Malus *suggest that both are functional. The three organellar *rps13 *genes in rosids provide a distinctive case of gene duplication involving the co-evolution of the nuclear and cytoplasmic genomes.

## Background

Gene duplication has been an ongoing process during eukaryotic evolution that has provided genetic raw material for the evolution of new gene functions that can lead to morphological and physiological novelty. Duplicated genes can undergo sequence divergence caused by positive selection or neutral drift [1-3] and divergence in expression patterns and function. Two common fates of retained duplicated genes are neofunctionalization – gain of a new function or expression pattern by one copy [4] and subfunctionalization – partitioning of ancestral function or expression pattern between both copies [5,6]. Plant genomes contain large numbers of duplicated genes, derived by polyploidy, segmental duplications, tandem duplications, and retroposition of cDNAs. Many duplicated genes in plant genomes have been preserved and undergone purifying selection, a few have undergone positive selection and functional diversification, and some have experienced subfunctionalization [7-10].

There are three genes that code for organellar ribosomal S13 genes among rosid species. Analysis of *rps13 *genes in the rosid species *Arabidopsis thaliana*, cotton (*Gossypium arboreum*), and soybean (*Glycine max*) revealed the presence of two expressed copies of *rps13 *in the nucleus that were derived by gene duplication [11,12]. Both *in vitro *and *in vivo *RPS13 protein import experiments indicated that one copy encodes the chloroplast-imported protein (nucp *rps13*) while the other encodes mitochondria-imported RPS13 (numit *rps13*) [11,12]. It was inferred that the missing mt *rps13 *gene product has been functionally replaced by the product of numit *rps13 *in a common ancestor of *Arabidopsis*, cotton, and legumes. Thus the function of numit *rps13 *has been modified after gene duplication, and one could argue that numit *rps13 *has gained a new function because it is operating in a new cellular context (the mitochondrial ribosome instead of the chloroplast ribosome). Subsequently mt *rps13 *was lost from mitochondrial DNA many times during the evolutionary history of rosids, as inferred from a Southern blot hybridization survey [13](see Additional File 1). Surprisingly, however, there were many species of rosids that do appear to retain *rps13 *in the mitochondrion, based on Southern blot hybridizations, but the gene in some species may not be intact or functional.

The organellar *rps13 *genes in rosids provide an intriguing system to study gene duplication because the subcellular location and site of action of numit RPS13 has changed after gene duplication from the chloroplast to the mitochondrion. We have studied sequence evolution of numit *rps13 *among rosids to determine what kinds of amino acid changes have taken place and where those amino acids are located in the tertiary structure, as well as to test the hypothesis that there has been adaptive evolution. Also we have examined the divergence of expression patterns between numit *rps13 *and nucp *rps13*. After finding intact and expressed numit *rps13 *and mt *rps13 *genes in *Malus *we tested the hypothesis that there has been expression partitioning of the two genes in different organ types and/or stress conditions to preserve both genes.

## Results

### Identification of numit *rps13 *in *Malus*, *Populus*, and *Citrus *and phylogenetic analysis

**Figure 1:**
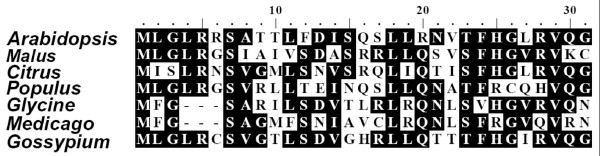
**Mitochondrial targeting sequence alignment of numit RPS13**. Predicted targeting presequences of numit RPS13 from seven rosid species are aligned. Identical amino acid residues are marked in white on the black background. Dots indicate gaps inserted to improve the alignment.

To study the evolution of numit *rps13 *sequences in the rosids, we identified sequences homologous to numit *rps13 *in *Malus, Populus*, and *Citrus *by BLAST searches of the NCBI expressed sequence tag (EST) database using the numit *rps13 *from *Arabidopsis *as a query. ESTs (see Materials and Methods) were aligned and the open reading frames were identified. The genes in each species are predicted with high probability to encode mitochondrial proteins by four prediction programs: MitoProt [14], TargetP v1.1 [15], Predotar v1.03 [16], and iPSORT [17]. Three of the four programs discriminate mitochondrial from chloroplast proteins and no support was obtained for chloroplast targeting. Alignment of the N-termini with mitochondrial targeting presequences from numit *rps13 *in other rosid species shows sequence conservation and indicates that they are homologous genes (Figure 1). Because numit *rps13 *in *Arabidopsis, Gossypium*, and *Glycine *have been experimentally shown to be imported into mitochondria but not chloroplasts (Adams et al., 2002), we infer that the products from the homologous genes in *Malus, Populus*, and *Citrus *also are targeted to mitochondria. We found numit *rps13 *sequences only in species belonging to the rosid lineage, suggesting that the gene duplication event that created numit *rps13 *likely occurred after the emergence of the rosid lineage. We did not find a numit *rps13 *sequence in *Vitis*, a group at the base of rosids [18,19], despite the mostly sequenced genome [20] and a large EST collection available at NCBI. We infer that the gene duplication event occurred at the base of the eurosids after separation from the Vitaceae lineage (see Additional File 1).

**Figure 2:**
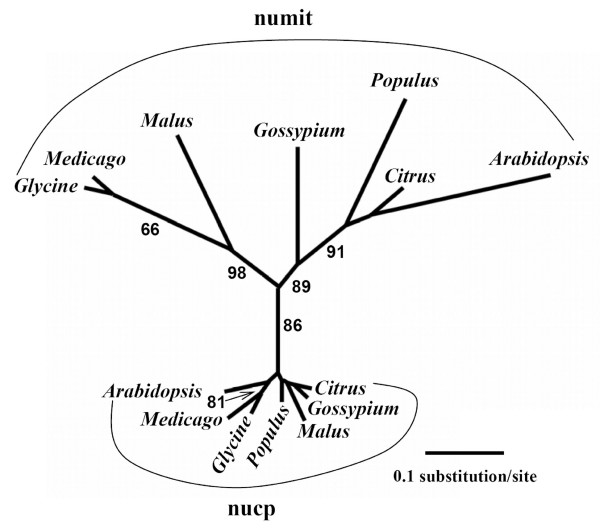
**Phylogenetic analysis of numit *rps13 *and nucp *rps13 *from rosid species**. Shown is an unrooted phylogram derived from maximum likelihood analyses of the first and second nucleotide positions. Bootstrap values from 100 replicates of ML analyses are labeled on the internodes.

Nucp *rps13 *sequences from seven rosid species were obtained from GenBank by BLAST searches using the previously characterized nucp *rps13 *from *Arabidopsis *[21] and aligned with the numit *rps13 *sequences. Phylogenetic analysis of the sequences verified the orthologous relationships of numit *rps13 *and nucp *rps13 *sequences (Figure 2). The phylogenetic relationships inferred from nucp *rps13 *and numit *rps13*, however, did not follow established relationships of rosid species [18]. For numit *rps13*, the positions of *Populus *and *Gossypium *in the tree are switched; for nucp *rps13 *the gene positions in the tree are completely scrambled. The lack of congruence between the gene trees and organismal phylogeny is probably due to the short sequences being analyzed (about 242 bp after excluding the third codon position). Alternatively, it might be caused by the complex history of multiple polyploidy events and subsequent loss of duplicated genes during rosid evolution. For example, after polyploidization the lineage containing legumes and *Malus *might have retained copy 1 while *Arabidopsis*, *Citrus*, *Gossypium *and *Populus *retained copy 2 (Figure 2). Comparisons of branch lengths showed that numit *rps13 *sequences have diverged rapidly with much longer branch lengths than the nucp *rps13 *sequences, suggesting there has been accelerated evolution of numit *rps13 *in each lineage, and consistent with results from Adams et al. [11] that included fewer species of rosids.

### Accelerated nucleotide substitution rates and positive selection on numit *rps13*

To better quantify the rate increase and to determine if both K_a _and K_s _(or just K_a_) are accelerated in numit *rps13 *sequences, the K_a_, K_s _values, and K_a_/K_s _ratios were compared between nucp *rps13 *and numit *rps13 *in the rosids. Significantly higher K_a _values and K_a_/K_s _ratios were observed in the numit *rps13 *genes than in the nucp *rps13 *genes (*P *< 0.05; Table 1; see Additional file 2). The K_a _values in the numit *rps13 *genes were 3–4 times greater than in the nucp *rps13 *genes. No significant difference in K_s _values, however, was found between the nucp *rps13 *and numit *rps13 *copies (*P *> 0.05; see Additional file 2). These results show that numit *rps13 *has been experiencing a considerably accelerated rate of non-synonymous substitutions compared with the nucp *rps13 *in all seven lineages of rosids.

**Table 1:** Comparison of K_a_/K_s _ratios between nucp *rps13 *and numit *rps13*.

Taxon	*Arabidopsis*	*Citrus*	*Glycine*	*Gossypium*	*Malus*	*Medicago*	*Populus*
nucp *rps13 *(0.06 ± 0.02)^a^							

*Arabidopsis*	-						
*Citrus*	0.019761	-					
*Glycine*	0.031462	0.111523	-				
*Gossypium*	0.045554	0.051540	0.076516	-			
*Malus*	0.037126	0.049357	0.081350	0.059803	-		
*Medicago*	0.040972	0.084293	0.059997	0.057981	0.085442	-	
*Populus*	0.048739	0.112004	0.062117	0.049504	0.061190	0.067945	-

numit *rps13 *(0.28 ± 0.08)							

*Arabidopsis*	-						
*Citrus*	0.276419	-					
*Glycine*	0.331700	0.253453	-				
*Gossypium*	0.257117	0.308765	0.299647	-			
*Malus*	0.299325	0.317365	0.238339	0.468253	-		
*Medicago*	0.267372	0.179926	0.032697	0.336763	0.273923	-	
*Populus*	0.382829	0.261000	0.241346	0.294584	0.301918	0.170266	-

^a^The mean and standard deviation are shown in parentheses.

Although simple pair-wise comparison of K_a _and K_s _analysis provides some insights into the accelerated amino acid substitution in numit *rps13*, branchwise estimation of K_a_, K_s _values, and K_a_/K_s _ratios can provide additional information such as positive selection and adaptive molecular evolution along certain branches and clades [22-26]. To detect if there has been positive selection acting on numit *rps13 *and nucp *rps13 *in different rosid species, site specific model analysis was conducted using PAML [23]. (Although synonymous and non-synonymous substitutions are abbreviated as d_S _and d_N _in PAML analysis, we use the common designations K_s _and K_a _to refer to synonymous and non-synonymous substitutions, respectively.) Both the *rps13 *tree (Figure 2) and the species tree that reflects our current understanding of rosid phylogeny [18] were used for detection of positive selection to determine if the tree topology influenced detection of positively selected sites, and no differences were found in this regard.

One ratio model (M0) shows that the K_a_/K_s _ratio of numit *rps13 *is about 4 times higher than nucp *rps13 *(Table 2), congruent with observations from simple K_a _and K_s _analysis. For each dataset, likelihood ratio tests (LRT) for detection of positive selection were examined using M7-M8 and M8a-M8 comparisons. For numit *rps13*, M8 is significantly better than M7 and M8a in numit *rps13 *(*P *< 0.05) and 2.3% of sites are inferred to undergo positive selection (Table 2), suggesting there has been positive selection acting on numit *rps13*. In addition, based on Bayes Empirical Bayes (BEB) analysis, two codon sites in numit *rps13 *were identified as showing strong positive selection (posterior probability > 0.8; Table 2). However, there is no evidence for sites under positive selection in nucp *rps13*. In nucp *rps13*, M8 is not significantly better than M7 and M8a (Table 2). These results indicate that strong positive selection acts on the evolution of numit *rps13*among the rosid species, particularly at codons 28 and 114.

**Table 2:** LRT statistics of site specific model for numit *rps13 *and nucp *rps13*.

Gene	Number of sequences	Tree Length^a^	K_s_^a^	K_a_^a^	K_a_/K_s_^a^	11th class from M8		M7-M8 comparison		M8a-M8 comparison		Selected positive sites
							
						p, %	K_a_/K_s_	2δL	*P *Value	2δL	*P *Value	
numit *rps13*	7	6.93	5.35	1.30	0.24	2.7	9.7400	9.0670	**0.0107^b^**	7.5048	**0.0031**	**28R**, 114R (*P *> 0.8)
nucp *rps13*	7	4.08	5.16	0.31	0.06	1.6	1.6268	4.7952	0.0909	0.8448	0.1790	None

^a^Estimated using M0 model in PAML.

^b^Boldface indicates the statistically significant difference (*P *< 0.05).

### Structural location of positively selected amino acids

**Figure 3:**
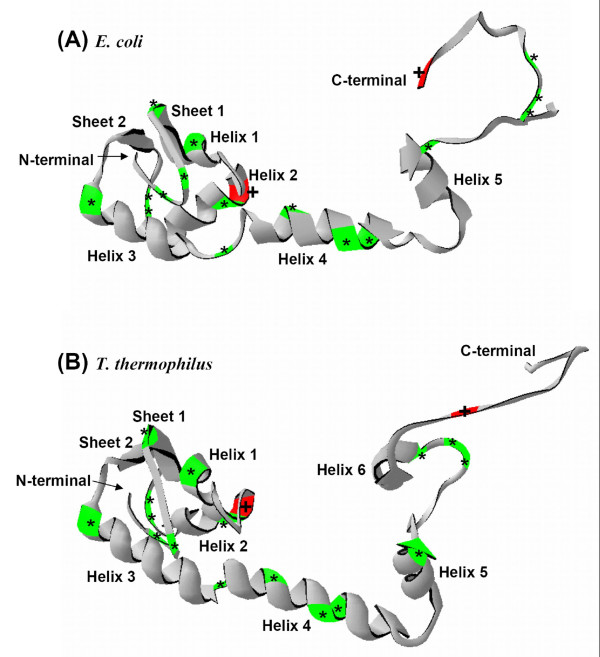
**Tertiary structure of numit RPS13**. The structures of RPS13 from *E. coli *(A) and *T. thermophilus *(B) are shown with amino acids from numit RPS13 plotted, as in the alignment (Figure 4). Red regions and plus signs indicate relative positions of positively selected sites. Green regions and asterisks indicate relative positions of amino acids in numit *Rps13 *in at least one rosid species that have mutated to the amino acid present in mt *rps13 *genes (Figure 4). Among them, sites 4 and 10 (Figure 4) are located in the coil region of the N-terminal end, site 15 is in the beginning of the first β-sheet, site 21 is in the first α-helix, site 32 is in the second α-helix, sites 42 and 43 are in the coil region between the second α-helix and the second β-sheet, site 53 are in the third α-helix, site 67 is in the coil region between the third α-helix and the fourth α-helix, sites 74, 79, and 80 are in the fourth α-helix, and sites 97, 107, 108, and 110 are in the C-terminal coil region.

**Figure 4:**
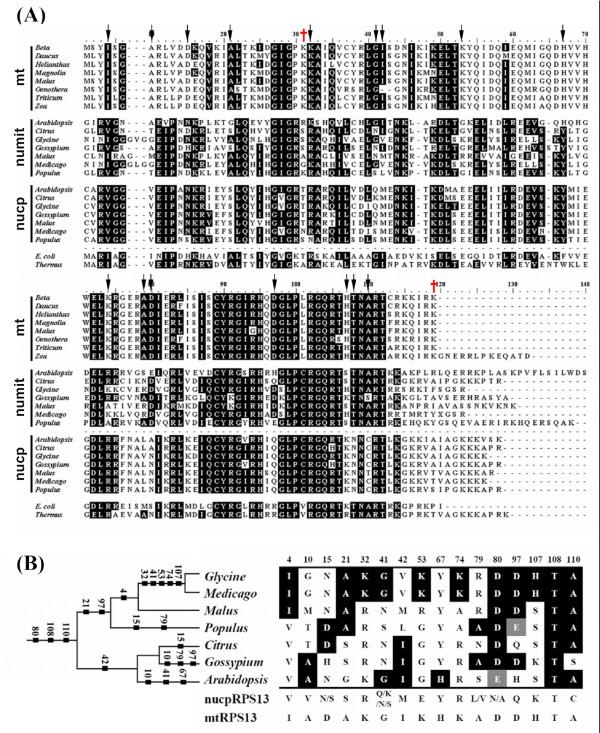
**Amino acid evolution in numit RPS13**. (A) Alignment of mt RPS13, numit RPS13, nucp RPS13 sequences, and RPS13 from *E. coli *and *Thermus thermophilis*. Amino acids in numit RPS13 that are identical to those in mt RPS13 or nucp RPS13 are shown on a black background. Dashes indicate gaps inserted to improve alignment. Red plus signs indicate positions of positively selected amino acids. Black arrows indicate amino acids in numit *rps13 *in at least one rosid species that have mutated to the amino acid present in all eight mt *rps13 *genes, summarized in panel B. Panel B also includes a phylogeny of the rosid species and our hypothesized evolutionary timing of each amino acid change. Numbers in panel B indicate positions in the alignment shown in panel A.

Relative locations of the two positively selected sites were plotted on the tertiary amino acid structures of RPS13 from *Escherichia coli *and *Thermus thermophilus *(Figure 3) to infer approximate locations in numit RPS13. Except for the C-terminal end which is longer and contains one more α-helix structure in *T. thermophilus*, the overall structure of RPS13 from *E. coli *and *T. thermophilus *are relatively similar (Figure 3). RPS13 residues at positions 28 and 114 in *E. coli *(28 and 116 in *T. thermophilus*) correspond to the positively selected sites in numit RPS13 based on the amino acid alignment (Figures 3, 4). Previous structural and functional studies of *Thermus *RPS13 showed that there are two structurally important regions where RPS13 interacts with the 16S rRNA [27-29]. One of them is the loop region between helix 1 and turn 1 (residues 22–25) [29]. One of the positively selected residues found in our analysis is close to that region. The second positively selected residue in numit *Rps13 *is located in the highly basic COOH-terminal extended region (Figure 3). This region is virtually devoid of secondary structure and found to interact with the 16S rRNA at the P-site and A-site (residues 116–120 for P-site and residues 120–122 for A-site) [27-29]. Positively selected sites in or near regions that interact with the 16S rRNA suggest that there has been functional refinement of numit RPS13 to interact better with the 16S rRNA.

### Amino acid changes in numit *rps13 *that increase identity to mt *rps13*

Because numit *rps13 *was derived from nucp *rps13 *and encodes a RPS13 protein that functions in the mitochondria, we were interested in determining if the numit RPS13 has become more like the mt RPS13 amino acid sequence. We determined if there have been mutations in the numit *rps13 *genes in any of the seven rosid species that change an amino acid to the residue that is present in mt RPS13. In our alignment of nucp RPS13, numit RPS13, and mt RPS13 across different plant species, sixteen sites were identified where numit RPS13 in one or more species has the same amino acid at the corresponding site in mt RPS13 from seven angiosperms, and the amino acid is different from the amino acid(s) present in nucp RPS13 (Figure 4A). The sixteen sites are relatively evenly distributed in the RPS13 tertiary structure (Figure 3). Although little is known about the exact functions of those regions, some of the mutations might help improve the function of numit RPS13 in the mitochondrial ribosome. We plotted the sixteen amino acid changes on the phylogeny of the seven rosid species to infer when they might have occurred (Figure 4B). Mutations are inferred to have occurred along most of the branches, suggesting continuous refinement of the numit *rps13 *sequence. We infer that three mutations occurred in the common ancestor of all the species, with a subsequent mutation at site 80 in *Arabidopsis *from D to E (a conservative substitution) and at site 110 in *Gossypium *from A to S, although scenarios with multiple recent mutations cannot be ruled out.

### Expression evolution of numit *rps13*

**Figure 5:**
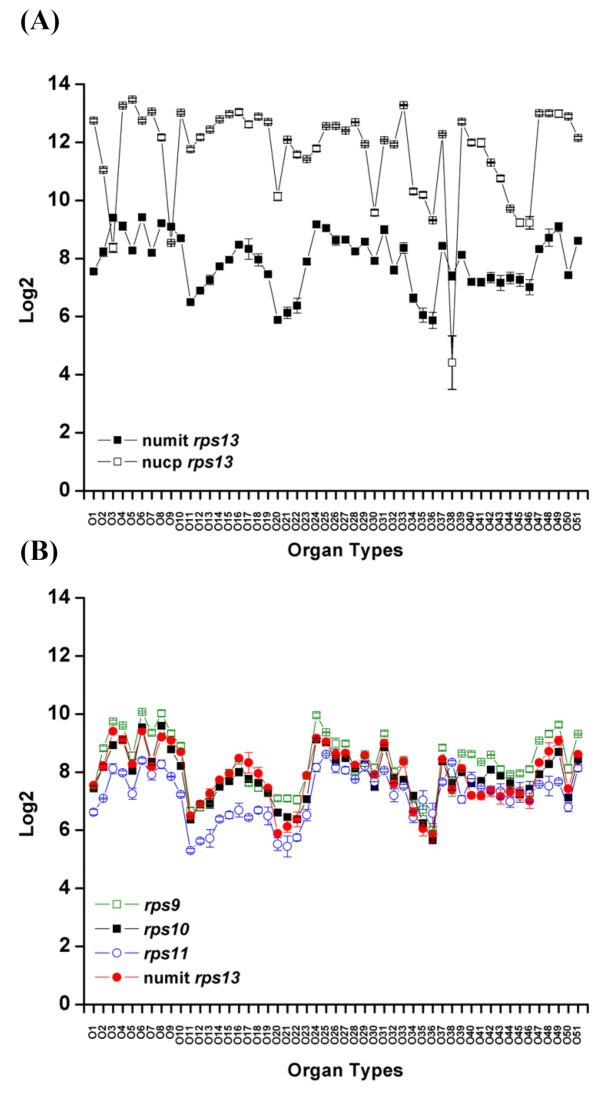
**Expression patterns of numit *rps13 *in *Arabidopsis thaliana***. Shown are graphs from ANOVA analysis of microarray data from 51 organs and developmental stages (see Methods). Organ types and developmental stages are listed in Additional File 3. Error bars show 3 biological replicates. The Y-axis indicates the expression level normalized by log2. (A) Numit *rps13 *compared with nucp *rps13*. (B) Numit *rps13 *compared with three other nuclear-encoded mitochondrial ribosomal protein genes, *rps9, rps10*, and *rps11 *[31].

Having studied sequence evolution of numit *rps13 *we next tested the hypothesis that there have been changes in expression patterns and levels of expression of numit *rps13 *relative to nucp *rps13*. Extensive microarray data are available for *Arabidopsis thaliana *including a single study that examined expression in 51 organs and developmental stages using the ATH1 array [30]. We analyzed expression data for numit *rps13 *and nucp *rps13 *using an ANOVA approach (see Methods). Nucp *rps13 *is expressed a higher level than numit *rps13 *in most organs (*P *< 0.05; Figure 5A; Additional file 3). Notable exceptions were roots, senescing leaves and pollen where numit *rps13 *is more highly expressed (*P *< 0.05; Figure 5A). When comparing expression levels of the two genes among organs, sometimes the levels of both genes go up or down together, but sometimes the levels go in opposite directions. Overall the organ-specific expression patterns between the two genes show both similarities and differences (R = 0.31, *P *< 0.05), depending on the organs compared.

To compare expression patterns of numit *rps13 *to other nuclear encoded ribosomal protein genes, we analyzed expression data for three other nuclear encoded mitochondrial ribosomal protein genes: *rps9, rps10*, and *rps11 *[31]. The expression levels and patterns of all four genes were highly similar (R = 0.73–0.94, *P *< 0.0001), although *rps11 *expression levels were lower in some organs (*P *< 0.05; Figure 5B). Thus numit *rps13 *has evolved an expression pattern similar to that of other nuclear-encoded genes for mitochondrial ribosomal proteins, and its expression has diverged from that of nucp *rps13*.

### *Malus *contains an expressed and RNA-edited copy of *rps13 *in the mitochondrion

Having studied the sequence and expression evolution of numit *rps13 *we next consider the fate of mt *rps13 *in rosids. A large number of rosids, including *Arabidopsis, Gossypium*, *Glycine*, and *Citrus *have lost mt *rps13*, as judged by DNA gel blot hybridization [13] (see Additional file 1), but some rosids retain *rps13 *in mitochondrial DNA. We identified a transcribed copy of *rps13 *in *Malus domestica *(apple) from BLAST searches of the NCBI EST database that is 90–92% identical with the *rps13 *gene in the mitochondrion of several eudicots. The sequence was derived from a study of ESTs in *Malus *[32]. A similar gene (97% identical) was found in *Prunus persica*, another member of the Rosaceae family. We evaluated the *rps13 *sequence from *Malus *for sites of C-to-U RNA editing by PCR amplifying and sequencing *rps13 *from genomic DNA and comparing the gDNA sequence to the EST sequences. C-to-U RNA editing plays an important role in the expression of plant mitochondrial genes to restore certain amino acids to those that are evolutionarily conserved [33]. Among angiosperm *rps13 *genes, nine possible edited sites have been identified (see Additional file 4). Four of those sites are already T's instead of C's in the genomic DNA sequence of mt *rps13 *from *Malus*, and thus RNA editing might be expected at five sites in the *Malus *cDNAs. No editing was observed in the two ESTs from *Malus*, and the EST from *Prunus *had editing at only one site (the 100th base). To verify the lack of RNA editing, we amplified and directly sequenced mt *rps13 *cDNAs from leaves and petals of *Malus*. Unexpectedly five RNA editing sites were discovered in both leaves and petals at five sites (see Additional file 4). After transcription, RNA editing converts codons from serine to leucine (the 26th, 56th, and 287th bases) and from arginine to cysteine (the 100th and 256th bases). The changes made by RNA editing would restore an evolutionarily conserved amino acid sequence and make the resulting protein likely to be functional, should the transcripts be translated.

### Co-expression of numit *rps13 *and mt *rps13 *in 14 different organ types and under five different stress conditions

**Figure 6:**
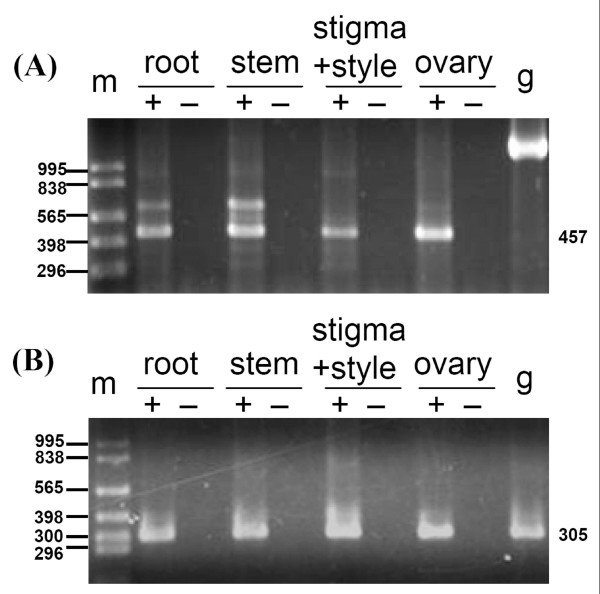
**Expression of numit *rps13 *and mt *rps13 *from in *Malus *in different organ types**. Plus signs indicate reactions containing reverse transcriptase (RT) and minus signs indicate reactions without RT. Abbreviations: coty, cotyledon; g, genomic; hypo, hypocotyl; m, marker. (A) A gel showing a subset of the RT-PCR products of numit *rps13 *(457 bp). Sequencing of a larger band in RT-PCR assays of numit *rps13 *revealed that it was due to non-specific primer binding, whereas the smaller band was the numit *rps13*. (B) A gel showing a subset of the RT-PCR products of mt *rps13 *(305 bp).

We conducted expression assays of numit *rps13 *and mt *rps13*, and RNA editing examination of mt *rps13*, in *Malus *to test the hypothesis that expression patterns have been partitioned between the two genes in different organ types. RT-PCR was performed with (RT+) or without (RT-) reverse transcriptase to check for DNA contamination (Figure 6). Transcripts of numit *rps13 *and mt *rps13 *were observed in roots (from seedlings), stems, stigmas + styles, and ovaries. Transcripts of these two genes were also observed in 10 additional organ types: hypocotyls, cotyledons, young leaves (from seedlings), mature leaves, peduncles, petals, seeds, sepals, stamens, and young fruit. Mt *rps13 *cDNAs were sequenced from each organ type to determine if any sites were edited. Different organ types in apple all showed the five RNA editing sites mentioned above. The results of the RT-PCR experiments show that both copies are co-expressed in all examined organ types.

Although both copies of *rps13 *are expressed in many organ types it is possible that there might be partitioning of expression between the two genes under stress conditions. We tested the hypothesis by examining the expression patterns of numit *rps13 *and mt *rps13 *under five different stresses including cold, dark, heat, salt, and water submersion treatments to determine if expression partitioning occurs under different environmental stresses. Apple seedlings were independently subjected to each of these five stresses (see Materials and Methods). Four organ types, including roots, hypocotyls, cotyledons, and leaves were examined for expression of numit *rps13 *and mt *rps13*. Based on RT-PCR results, transcripts of numit *rps13 *and mt *rps13 *were observed under each of the five stress conditions: cold, dark, heat, salt, and water submersion treatments. Sequencing of RT-PCR products showed that the transcripts were RNA edited at 5 sites, although in some cases there was partial editing at one or more sites.

**Figure 7:**
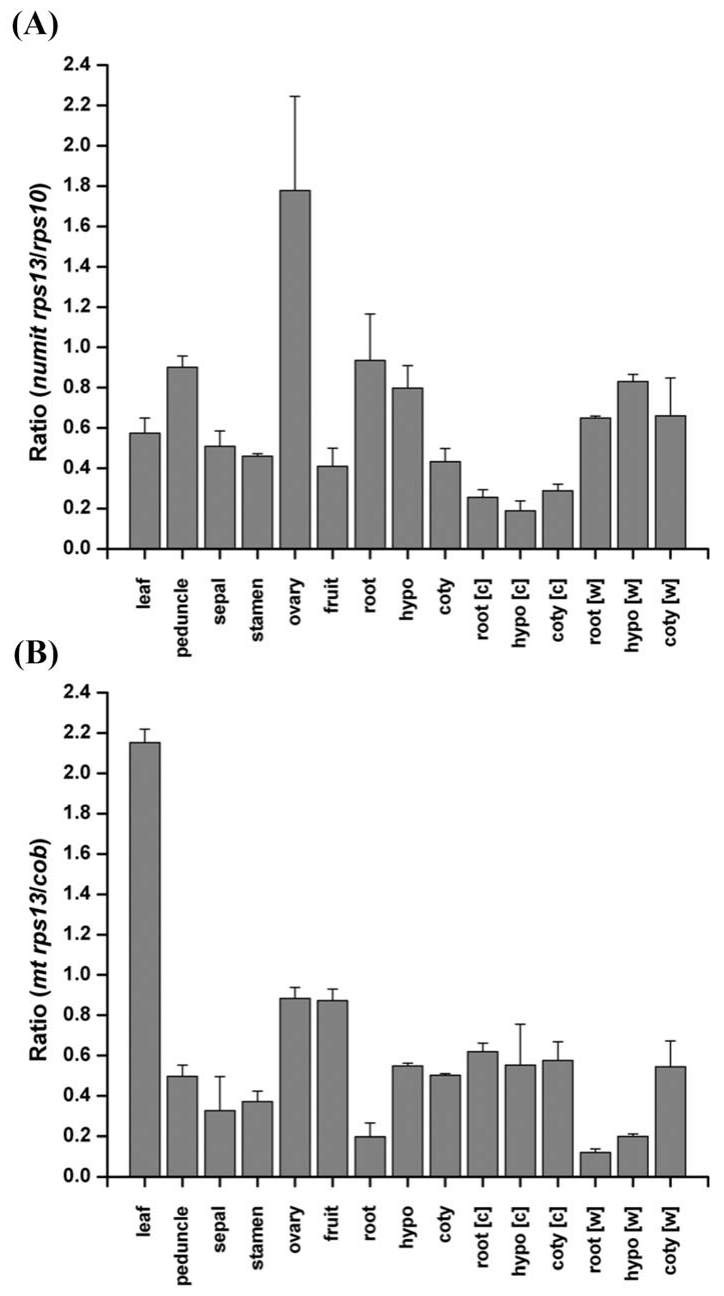
**qRT-PCR of numit *rps13 *and mt *rps13 *in *Malus***. (A) Ratios of numit rps13 to rps10 transcripts. (B) Ratios of mt rps13 to cob transcripts. Error bars show variation from two replicates.

We have shown transcription of both numit *rps13 *and mt *rps13 *in many organs and under several stress conditions. However it is possible that transcripts from one gene might be present at low levels. PCR of cDNA template (RT-PCR) is not a quantitative technique and low levels of transcripts might not be distinguishable from high levels. To determine if abundant levels of steady-state transcripts are derived from both genes, we used real-time PCR (qRT-PCR) to assay transcript levels of numit *rps13 *and mt *rps13*. We compared levels of numit *rps13 *to those of another nuclear gene for a mitochondrial ribosomal protein, *rps10 *(for ribosomal protein S10), and we compared levels of mt *rps13 *to those of another mitochondrial gene, *cob *(for cytochrome b). We assayed expression levels in a subset of 9 organs and two stress conditions that were assayed above. Numit *rps13 *transcripts were present at or above the levels of *rps10 *in ovaries and roots, and most organs showed at least half as many transcripts from numit *rps13 *as *rps10 *(Figure 7A). Cold stress was an exception, where the numit *rps13*/*rps10 *ratios were less than 0.4 (Figure 7A). Mt *rps13 *transcripts were present at slightly over twice the levels of *cob *transcripts in leaves (Figure 7B). The mt *rps13*/*cob *transcript ratio was above 0.5 in most other organs and under cold stress. Overall there is evidence for relatively high levels of steady-state transcripts from numit *rps13 *and mt *rps13 *in most organs.

### Purifying selection acting on numit *rps13*, nucp *rps13*, and mt *rps13 *in *Malus*

Detecting purifying selection, neutral evolution, and positive selection on each of the three *rps13 *genes would provide insights into the selective forces at work and could indicate if one copy is a pseudogene. A K_a_/K_s _ratio less than one would indicate purifying selection and be evidence for a functional gene, and a K_a_/K_s _ratio of about one would be evidence of neutral evolution and thus probably pseudogenization. The branch specific model in PAML was used to detect adaptive molecular evolution in numit *rps13*, nucp *rps13*, and mt *rps13 *for the *Malus *branches by comparing with other plant species. For each LRT, M1 is the model assuming neutral evolution among sites, MA_test1 _assumes that there are positively selected amino acid sites for the foreground lineage, and MA_test2_, is used to test if the detection of positive selection is an artifact. LRTs showed that MA_test1 _is not significantly better than M1 and MA_test2 _in *Malus *numit *rps13 *and *Malus *mt *rps13*, indicating that there is no positive selection for these two branches (Table 3). For nucp *rps13*, MA_test1 _is significantly better than M1, but not better than MA_test2 _(Table 3), suggesting that there is no positive selection in nucp *rps13*. In addition, branch specific models can provide detailed information about how many sites are undergoing purifying selection, neutral evolution, and positive selection. For numit *rps13*, 65% of the sites are under purifying selection with K_a_/K_s _less than one, 28% of sites are under neutral evolution with K_a_/K_s _close to one, and 7% of sites are under positive selection with K_a_/K_s _greater than one (Table 3) without statistical support by LRTs. For nucp *rps13*, 90% of sites are under purifying selection, 7% of sites are under neutral selection, and 3% of sites are under positive selection (Table 3) although the positively selected sites are not supported by LRTs. For mt *rps13*, 66% of sites are under purifying selection and 34% of sites are under neutral selection (Table 3). The number of sites experiencing each type of selection in numit *rps13 *in *Malus *is similar to numit *rps13 *in other rosids that do not have mt *rps13 *(Table 4). Overall our results reveal that the three ribosomal protein S13 genes in *Malus *are undergoing higher purifying selection than neutral evolution, suggesting that their functionality is maintained by purifying selection.

**Table 3:** LRT statistics of branch specific model for *Malus *branch.

Gene	Parameters from MA_test1_	M1-MA_test1 _comparison		MA_test2_-MA_test1 _comparison		Selected positive sites
			
		2δL	*P *Value	2δL	*P *Value	
numit *rps13*	p_0 _= 0.6485, p_1 _= 0.2817, (p_2 _+ p_3 _= 0.0698) ω_0 _= 0.12, ω_1 _= 1, ω_2 _= 112.98	1.7396	0.4190	1.2140	0.2705	31H, 32Q, 117R (0.5 <*P *< 0.8)
nucp *rps13*	p_0 _= 0.9005, p_1 _= 0.0733, (p_2 _+ p_3 _= 0.0262) ω_0 _= 0.04, ω_1 _= 1, ω_2 _= 96.7	9.7274	**0.0077^a^**	3.5026	0.0613	**32Q, 105C**
mt *rps13*	p_0 _= 0.6646, p_1 _= 0.3354, (p2 + p3 = 0) ω_0 _= 0, ω_1 _= 1, ω_2 _= 1	0	1	0	1	None

^a^Boldface indicates the statistically significant difference (*P *< 0.05).

**Table 4:** Selection on numit RPS13 in seven rosid species.

Taxon	Purifying Selection (%, K_a_/K_s _< 1)^a^	Neutral Evolution (%, K_a_/K_s _= 1)^a^	Positive Selection (%, K_a_/K_s _> 1)^a^	M1-MA_test1 _comparison		MA_test2_-MA_test1 _comparison		Selected positive sites
					
				2δL	*P *Value	2δL	*P *Value	
*Malus*	65	28	7 (120.33)^b^	1.7396	0.4190	1.2140	0.2705	31H, 32Q, 117R
*Citrus*	65	30	5 (154.19)	4.3230	0.1152	3.9182	**0.0478^c^**	50K, 92H
*Glycine*	67	33	0	0	1	0	1	35C
*Medicago*	67	33	0	0	1	0	1	None
*Gossypium*	64	36	0	0.6314	0.7293	0.0024	0.9609	108K
*Populus*	66	30	4 (18.05)	3.6624	0.1603	3.4746	0.0623	39I, 88S, 102S
*Arabidopsis*	52	48	0	6.1770	**0.0456**	0	1	18G, 36H, 60G, 83D

^a^Estimated using MA_test1_.

^b^Bracket indicates K_a_/K_s _ratio. If K_a_/K_s _ratio is close to one for the third and fourth class of MA_test1 _without statistical support to be greater than one, it will be counted into the category of neutral evolution.

^c^Boldface indicates a statistically significant difference (*P *< 0.05).

## Discussion

### Continuous accelerated evolution and molecular adaptation of numit *rps13 *among rosids

After gene duplication some retained duplicated genes undergo asymmetric rate divergence and the faster evolving copy experiences relaxed constraint or positive selection [34,35]. The increased K_a _rate, K_a_/K_s _ratio, and positively selected sites detected in numit *rps13*, compared with nucp *rps13*, across different rosid species show there has been accelerated sequence evolution of numit *rps13*. The increased K_a _rate is likely to be correlated with the modified function of numit *rps13 *– from encoding a chloroplast ribosomal protein to a mitochondrial ribosomal protein. Some amino acid sequence changes were probably necessary for numit RPS13 to interact well with other proteins in the mitochondrial ribosome. The increased rate of non-synonymous substitutions is a continuing process instead of there being a burst of sequence change upon formation of numit *rps13 *followed by a prolonged period of lower rates of non-synonymous substitutions. The continued higher K_a _rate, and the two positively selected codons in numit *rps13 *near the positions where RPS13 interacts with 16S RNA domain, show that the sequence, and suggest that the function, of numit RPS13 in mitochondria is continuing to be refined. Site-specific positive selection has been detected in other duplicated genes [36-38]. For example, in duplicated *AP3 *and *PI *genes there has been functional diversification driven by positive selection acting on different sites within a functional domain involved in heterodimerization [39].

The sixteen amino acids in one or more rosid species that have changed to the amino acid present in mt RPS13 (Figure 4) indicate that numit RPS13 is becoming more like mt RPS13. We propose that this is a type of convergent sequence evolution of numit *rps13 *that possibly improves the function of numit RPS13 in the ribosome. The amino acid changes appear to have been taking place continuously during rosid evolution (Figure 4B), with the largest number (five) having occurred on the branch leading to the legumes *Glycine *and *Medicago*. We speculate that some of the amino acid changes that have taken place along the terminal or subterminal branches of the tree might have allowed numit *rps13 *to be selected for, over mt *rps13*, and allowed multiple independent losses of mt *rps13 *in different lineages (see Additional file 1). In species where mt *rps13 *has been lost the product of numit *rps13 *presumably functions as well as, or perhaps better than, mt *rps13 *or else there would have been selection for retention of mt *rps13*. Overall the adaptive and convergent evolutionary forces that seem to be acting on numit *rps13 *have continued during rosid evolution instead of being factors only soon after gene duplication in a common ancestor of most rosids.

### Expression evolution of numit *rps13 *and nucp *rps13 *in *Arabidopsis*

Expression patterns of some duplicated genes have been shown to evolve in a divergent and sometimes asymmetric manner [40-42]. For example, many of the genes derived by an ancient polyploidy event in the *Arabidopsis *lineage have undergone considerable expression divergence [43-45], some of which may have been asymmetric between the two duplicates. Considering that numit *rps13 *and nucp *rps13 *show asymmetric divergence in sequence, and different subcellular locations of their protein products, we predicted that they also would show asymmetric divergence in expression, with the expression pattern of numit *rps13 *being similar to that of other nuclear-encoded genes for mitochondrial ribosomal proteins. Numit *rps13 *and nucp *rps13 *did show some differences in expression patterns although the differences were only dramatic in roots, senescing leaves, and pollen. Numit *rps13 *has evolved a similar expression pattern to three other genes for mitochondrial ribosomal proteins, perhaps by gaining regulatory elements from another gene for a mitochondrial protein, as have several mitochondrial genes that have been transferred to the nucleus [46]. Alternatively there may have been mutations in the regulatory elements of numit *rps13 *soon after gene duplication that produced an expression pattern similar to that of other genes for mitochondrial ribosomal proteins. Considering that numit *rps13 *was formed in a common ancestor of most rosids, distinguishing between the above possibilities about the origin of its regulatory elements is impossible. Overall numit *rps13 *and nucp *rps13 *add to the growing number of duplicated genes reported in *Arabidopsis thaliana *that have experienced divergence in expression patterns.

### Co-expression of numit *rps13 *and mt *rps13 *in *Malus*

We have shown that numit *rps13 *and mt *rps13 *in *Malus *are both transcribed in 14 different organ types and under five abiotic stress conditions, mt *rps13 *is RNA edited at sites to make the transcripts contain an evolutionarily conserved sequence, and abundant steady-state transcripts from both genes are present in a variety of organs. Both genes are experiencing purifying selection. Taken together, the data obtained in this study indicate that it is likely that both numit *rps13 *and mt *rps13 *in *Malus *are functional genes and not pseudogenes. However there is the possibility, albeit unlikely, that transcripts from one gene (particularly mt *rps13*) might not be translated. Even if both proteins are present, there is the possibility that only one is assembled into mitochondrial ribosomes, and that process could vary among organs and environmental conditions. Showing that both numit RPS13 and mt RPS13 proteins are assembled into mitochondrial ribosomes, especially in a variety of organ types and under various environmental conditions, is beyond the scope of the current evolutionary study.

Although we have provided evidence for the functionality of the gene products from numit *rps13 *and mt *rps13 *in *Malus*, might numit *rps13 *have experienced functional reversion to encode a chloroplast protein? Several lines of evidence do not support that possibility. The mitochondrial targeting presequence of numit RPS13 is similar to those of numit RPS13 in *Arabidopsis, Gossypium*, and *Glycine *(Figure 1), each of which was experimentally determined to be imported into mitochondria but not chloroplasts [11,12]. Numit *rps13 *in *Malus *does not have a greatly accelerated rate of sequence evolution or show more sites under positive selection, compared with numit *rps13 *in other rosids, as might be expected if its product now functions in the chloroplast. Instead purifying selection is operating on numit *rps13 *in *Malus*. Finally, there is no evidence to suggest that nucp *rps13 *in *Malus *is a pseudogene and indeed the gene is experiencing strong purifying selection.

Co-expression of numit *rps13 *and mt *rps13 *genes in *Malus *contrasts with the *atp9 *genes in *Neurospora crassa *where the nuclear and mitochondrial copies are expressed during different stages of the life cycle [47] and expression has been partitioned between the two genes. Presumably the nuclear copy of *atp9 *was derived from transfer of the mitochondrial gene to the nucleus in an ancestor of *Neurospora*, and the current availability of several ascomycete genome sequences could shed light on the evolutionary timing of the gene transfer. Only three other cases of co-expression of nuclear and mitochondrial genes have been reported, to our knowledge, including *cox2 *genes in multiple legume species within the Phaseoleae tribe [48], *rpl5 *genes in wheat (*Triticum aestivum*) [49], and *sdh4 *genes in *Populus *[50]. Those genes contrast to *rps13 *in rosids because the nuclear copies were derived by transfer of the mitochondrial gene to the nucleus.

Considering that numit *rps13 *was created by gene duplication in the common ancestor of most rosids, co-expression of mt *rps13 *and numit *rps13 *in *Malus *has presumably occurred for a long period of evolutionary time. Other cases of co-expression of nuclear and mitochondrial genes in plants represent evolutionarily recent gene transfers to the nucleus. Indeed following transfer of a mitochondrial gene to the nucleus the mitochondrial copy is often lost in a relatively short amount of time [13]. Thus it was unexpected that numit *rps13 *and mt *rps13 *have been preserved without partitioning of expression patterns. Why would both copies continue to be retained and expressed? One possibility is that there is partitioning of expression in organs, tissues, or cell types, or under environmental conditions that were not examined in this study. Another possibility is that, despite the considerable level of sequence divergence between numit *rps13 *and mt *rps13 *(about 42% identity), the product of either gene functions well in the mitochondrial ribosomes of *Malus*. That possibility is supported by the fact that some rosids (such as *Arabidopsis, Gossypium, Glycine*, and *Citrus*) have only the numit *rps13 *and non-rosid angiosperms have only mt *rps13*. Another possibility is that mutations have not occurred in numit *rps13 *from *Malus *that cause it to be selected for over mt *rps13*, as presumably occurred in other lineages of rosids that have lost mt *rps13*. In this regard it is notable that there are no amino acid changes in numit RPS13 to the residue present in mt RPS13 that occurred along the terminal branch leading to *Malus *(Figure 4B), unlike all of the other terminal branches on the tree leading to species that have lost mt *rps13*.

## Conclusion

The accelerated rate of amino acid evolution, positive selection on specific sites, and amino acid changes to the residue present in mt RPS13 provide evidence that numit *rps13 *genes in rosids have experienced adaptive evolution and convergent sequence evolution with mt *rps13*. Numit *rps13 *provides an example of a gene that has experienced site-specific positive selection while the gene as a whole has been under purifying selection. Expression patterns of numit *rps13 *have diverged to become similar to those of other genes for mitochondrial ribosomal proteins. *Malus *contains intact genes for both numit *rps13 *and mt *rps13 *that are transcribed in a range of organs and under several abiotic stresses. Abundant levels of steady state transcripts from both numit *rps13 *and mt *rps13 *in *Malus *in a variety of organ types, as well as purifying selection acting on both genes, suggest that both genes are functional and not pseudogenes. The three organellar *rps13 *genes in rosids provide a distinctive case of gene duplication involving the co-evolution of the nuclear and cytoplasmic genomes.

## Methods

### Database searches

The following expressed sequence tags (EST) for numit *rps13 *were obtained from GenBank by BLAST searches using numit *rps13 *from *Arabidopsis thaliana *(DR380621) as a query: *Citrus sinensis *(CX672493), *Malus domestica *(DR995890, CN494589, CN925904, and CX023021), *Populus trichocarpa *(DT496554), *Medicago truncatula *(BI272420), *Glycine max *(BM188020), and *Gossypium hirsutum *(DW488419). The following ESTs for nucp *rps13 *were obtained from GenBank by BLAST searches using nucp *rps13 *from *Arabidopsis thaliana *(DR367899) as a query: *Citrus sinensis *(CX053776), *Glycine max *(EH261685), *Gossypium hirsutum *(DW226103), *Malus domestica *(DT000985), *Medicago truncatula *(BI265445) and *Populus trichocarpa *(DT486949). Mt *rps13 *ESTs from *Malus *(CN871057 and CN875489) and *Prunus persica *(AJ873687) were identified by BLAST searches of GenBank using mt *rps13 *from *Beta vulgaris *as a query. Mt *rps13 *genes used in this study include: *Triticum aestivum *(Y00520), *Zea mays *(AF079549), *Magnolia *spp. (Z49799), *Helianthus annuus *(AJ243789), *Daucus carota *(X54417), *Oenothera berteriana *(X54416), *Beta vulgaris *(DQ381464) and *Marchantia polymorpha *(M68929).

### Sequence alignment/analysis and phylogenetic analysis

Sequence alignment was done using transAlign, which aligns protein-coding DNA sequence based on the alignment of amino acids [51]. Aligned sequences were refined with BioEdit [52] for further phylogenetic and codon substitution analysis (see Additional file 5). For phylogenetic analysis, maximum likelihood (ML) analysis was conducted with MultiPhyl v1.0.6 [53] with SPR (subtree pruning and regrafting) branch swapping. The optimal evolutionary model selected for ML was K81uf+I+G (Kimura three parameter with unequal base frequencies + proportion of sites + gamma distribution) using the following parameters: assumed nucleotide frequencies A = 0.36736, C = 0.20950, G = 0.27698, T = 0.14616; expected transition/transversion ratio = 0.94; expected pyrimidine transition/purine transition ratio = 0.30; proportion sites assumed to be invariable = 0.29; rates for variable sites assumed to follow the gamma distribution with shape parameter = 4.28. Bootstrapping was performed using MultiPhyl v1.0.6 with neighbor-joining algorithm and 100 replicates [53].

### K_a_/K_s _analyses and likelihood ratio tests (LRT) for positive selection

Nonsynonymous (K_a_) and synonymous (K_s_) nucleotide substitution rates were calculated by using program yn00 in PAML v3.15 [23] (see Additional file 2). The *t*-test was used for testing for significant differences of K_a_, K_s_, and K_a_/K_s _ratios between the nucp *rps13 *and numit *rps13 *cDNA sequences. The statistically significant level was set at 95%. The statistical analysis in this study was implemented by using the statistical package R [54].

For detection of positive selection, codon-based analysis was implemented using codeml in PAML v3.15 [23]. The mature coding region, without the mitochondrial targeting presequences, was included in our analysis. Site specific models were used for testing positive selection on numit *rps13 *and nucp *rps13 *[25]. Two LRTs were used for the detection of positive selection: M7-M8 and M8a-M8 [22,25,26]. Because comparison of M7 and M8 provides a more powerful test of positive selection and has less sensitivity to large evolutionary distances and G+C content than comparison of M1 and M2 [55,56], only comparison of M7 and M8 was used for the detection of positive selection in this study. M7 and M8a models are the null models without positive selection (no codon with K_a_/K_s _> 1) and the M8 model is the alternative model with positive selection. Branch specific model was used to test if there has been positive selection for *Malus *branch in numit *rps13*, nucp *rps13*, and mt *rps13 *[25]. For this analysis, the branch which we are interested in testing positive selection was assigned as foreground lineage. Therefore, *Malus *branch in numit *rps13*, nucp *rps13*, and mt *rps13 *were set as foreground lineage and the rest of other rosid species were designated as background lineage. For branch specific model, two LRTs were used for detection of positive selection as follows: M1-Model A test1 (MA_test1_) and Model A test2 (MA_test2_)-MA_test1 _[25,26]. M1 and MA_test2 _models are the null hypothesis without positive selection (no codon with K_a_/K_s _> 1) and the MA_test1 _model is the alternative selection with positive selection. For site-specific and branch-specific models used for detection of positive selection, M8a is more stringent than M7 and MA_test2 _is more stringent than M1 because their ω_2 _(K_a_/K_s_) is fixed at 1. For all LRTs, the first model is simpler than the second one, with fewer parameters and poor fit to the data. Therefore, the first model has a lower maximum likelihood index. To test if there is statistically better maximum likelihood for the second model, twice difference of log maximum likelihood values between the two compared models [2δL = 2(Ln2-Ln1), where Ln1 and Ln2 represent for log of maximum likelihood value in the first model and the second model] was compared against χ^2 ^distribution. The degrees of freedom (d.f.) equals to the additional parameters used in more complex model. The d.f. is 2 for M7-M8 and M1-MA_test1_, while the d.f. is 1 for M8a-M8 and MA_test2_-MA_test1_. However, it was argued that the appropriate model comparison for M8a-M8 would be to use 50:50 mixture of d.f. = 0 and d.f. = 1 [22]. In this study, the calculation of *P *value for M8a-M8 was followed as described in Kapralov and Filatov [57]. *P *value was first obtained using d.f. = 1 and then divided by 2. Bayes empirical bayes (BEB) approach was used to determine positive selected sites in M8 model and MA_test1 _model. In BEB analysis, the posterior probabilities were calculated to select the codon with K_a_/K_s _greater than 1. Because BEB analysis is more powerful to detect positive selection than naive empirical Bayes (NEB) analysis [58], only BEB analysis was considered in this study.

### Structural analysis of RPS13

Using first approach mode in SWISS-MODEL [59], nucp, numit, and mt RPS13 amino acid sequences in *Malus *were selected to search for a suitable template for further structural analysis. Based on the results, RPS13 of *Escherichia coli *and *Thermus thermophilus *were chosen for the following structural analysis. RPS13 structural data file for *E. coli *(PDB ID: 2gy9M) [60] and *T. thermophilus *(PDB ID: 1fjgM) [27] were obtained from RCSB Protein Data Bank [61]. Location and property of each amino acid in *E. coli *and *T. thermophilus *RPS13 was analyzed using DeepView-Swiss-PdbViewer v.3.7 [59]. Selected positive sites obtained by BEB analysis and sites shared with similarity between numit RPS13 and mt RPS13 were plotted in the relative position of RPS13 in *E. coli *and *T. thermophilus *according to amino acid alignment (Figure 4).

### Microarray data analysis

Raw Affymetrix ATH1 microarray data [30] were downloaded from the TAIR website (TAIR accession number: ME00319)[62]. Raw data were processed and normalized based on the GC-RMA method [63]. The expression values were converted into log2 numbers by only considering values of perfect-match probes [63]. Normalization and analysis of the microarray data were implemented using Bioconductor. Pearson correlation analysis was conducted to statistically compare the similarity of expression profile among *rps9*, *rps10*, *rps11*, and numit *rps13*, and between numit *rps13 *and nucp *rps13*. The correlation coefficient (R) values correspond to the similarity of the expression profile between two genes. A one-way ANOVA with Bonferroni post hoc tests was used to test if there is a significant expression difference between two different genes or any two different organ types. All statistical analyses were implemented using statistical package R [54]. The statistically significant level was set at 95%.

### Plant materials and abiotic stress treatments

Several organ types, including stems, leaves, peduncles, sepals, petals, stamens, stigmas+styles, and young fruit of apple (*Malus domestica *Borkh.) were collected from the University of British Columbia's Botanical Garden. Seedlings were used for abiotic stress experiments. Prior to sowing, apple seeds were soaked in distilled water at 4°C for 6 weeks for vernalization. Seedlings were cultivated in a peat-vermiculite soil mixture under fixed day/night (16 h day/8 h night) and temperature of 20–23°C. After germination and emergence from the soil for 7 days, the plants were subjected to five different abiotic stresses: 4°C for 7 days (cold treatment), 37–40°C for 12 hours (heat stress), 100 mM NaCl solution for 7 days (salt treatment), submerged in distilled water for 4 days (water submersion treatment), and dark for 7 days (dark treatment). After stress treatments, roots, hypocotyls, cotyledons, and leaves were collected and immediately frozen in liquid nitrogen and stored at -80°C until nucleic acid extraction.

### Nucleic acid extraction, gene amplification, and sequencing

Genomic DNA extraction was done using the DNeasy Plant Mini Kit (Qiagen) following the manufacturer's protocol. Total RNA extraction was performed as described previously [64]. The extracted nucleic acid concentrations and purities were determined by using a NanoDrop spectrophotometer. The quality was checked by running on 1.5% agarose gels. Before reverse transcription, 3 μg of RNA (500 μg/μl) was treated with 1 unit of DNaseI (New England Biolabs) and incubated at 37°C for 30 min twice. Then 4 μg of DNase-treated RNA was reverse-transcribed by using M-MLV reverse transcriptase (Invitrogen). The reverse transcription conditions were 25°C for 10 min, 37°C for 60 min, and 70°C for 15 min. Finally, the reverse-transcribed samples were treated with RNase (Invitrogen) at 37°C for 20 min.

The *rps13 *genes were amplified from genomic DNA and cDNA by polymerase chain reaction (PCR) using gene-specific primers (see Additional file 6). The PCR was performed in a reaction mixture (10 μl) consisting of 4.88 μl of ddH_2_O, 1 μl of genomic DNA/cDNA solution, 1 μl of PCR buffer, 1 μl of 2.5 mM MgCl_2 _solution, 1 μl of 0.2 mM dNTPs, 0.5 μl of 0.4 μM each primer, and 0.12 units of Taq DNA Polymerase (Sigma). The PCR conditions were 96°C for 4 min, and 30 cycles of 96°C for 40 s, 60°C for 40 s, 72°C for 1 min, and 72°C for 10 min. The PCR products were run on a 1.5% agarose gel and extracted from the gel using QIAquick Gel Extraction Kit (Qiagen). PCR products amplified from genomic DNA and cDNA were sequenced directly. The sequencing was performed in a reaction mixture containing 0.4 μl of ABI BigDye Version 3.1 (Applied Biosystems), 3.6 μl of BigDye buffer, 5.5 μl of 50 ng template, and 0.5 μl of 0.4 μM forward or reverse primer. The sequencing reaction was carried out with the following program: 1 min at 96°C, and 25 cycle of 10 s at 96°C, 5 s at 50°C, and 4 min at 60°C. The sequencing products were run on an ABI 377 DNA Sequencer (Applied Biosystems) at the UBC Centre for Plant Research, or on an ABI 3730 Sequencer at the Nucleic Acids Protein Service unit at UBC. The GenBank accession number for mt *rps13 *in *Malus*, including information about RNA editing sites, is [GenBank: EU084692].

### Real-time qRT-PCR

Quantitative real-time RT-PCR was performed with a BioRad iQ5 system using SYBR green master mix (BioRad) following the manufacturer's instructions, except that 25μl total reaction volumes were used. The PCR conditions were 96°C for 3 min, and then 35 cycles of 96°C for 10 s, 58°C for 30 s, and 72°C for 30 s. Gene-specific primers are listed in Additional file 6. For each sample, two technical replicates were performed. Reactions for a standard curve were run with each set of experimental reactions. After the completion of PCR, the melting curves were analyzed to distinguish the true product from artifacts such as primer dimers. The iQ5 software and Microsoft Excel were used for data analysis. Normalization was done using the actin gene *ACT2*. GenBank accession numbers for sequences used to design primers are: *Malus ACT2*: CN903171, CN902302, and N917499; *Malus cob*: CN872477, CN856986, CN856316; *Malus rps10*: CV882925.

## Abbreviations

BEB, Bayes Empirical Bayes analysis; EST, expressed sequence tag; LRT, likelihood ratio test; ML, maximum likelihood; NCBI, National Center for Biotechnology Information; qRT-PCR, quantitative real-time polymerase chain reaction; RT-PCR, reverse transcription-polymerase chain reaction

## Authors' contributions

SLL planned and carried out the experiments and analyses, and wrote the manuscript. KA conceived the study, helped with the experimental design, and wrote the manuscript. Both authors read and approved of the final manuscript.

## Supplementary Material

Additional file 1 **Mt *rps13 *distribution among rosids**. The figure shows losses of mt *rps13 *among rosids in a phylogenetic context, and the inferred timing of numit *rps13 *formation by gene duplication. Click here for file

Additional file 2 **Pairwise K_a _and K_s _analyses**. The table shows the results of K_a _analysis and K_s _analysis for numit *rps13 *and nucp *rps13 *in seven rosid species. Click here for file

Additional file 3 **Microarray data legend**. The table lists the organs and developmental stages from *Arabidopsis thaliana *for which microarray data were analyzed. Click here for file

Additional file 4 **RNA editing of mt *rps13 *in *Malus *compared with other flowering plants**. The figure shows an alignment of mt *rps13 *from several land plants, indicating the positions of RNA editing sites in *Malus*, and a list of mt *rps13 *RNA editing sites from several angiosperms. Click here for file

Additional file 5 **Sequence alignment**. The figure shows an alignment of numit *rps13*, nucp *rps13*, and cDNAs of mt *rps13 *for taxa analyzed in this study. The targeting sequences at the 5' end of the numit *rps13 *and nucp *rps13 *genes do not align with each other and were excluded from K_a_/K_s _analysis. Click here for file

Additional file 6 **RT-PCR primers**. The table lists the primers used for RT-PCR and qRT-PCR experiments. Click here for file
