# Supplementary material for: Molecular adaptation and expression evolution following duplication of genes for organellar ribosomal protein S13 in rosids
Source: BMC Evol Biol. 2008 Jan 26;8:25. doi: 10.1186/1471-2148-8-25 (PMC2258280; doi:10.1186/1471-2148-8-25)
Supplement: Additional file 1 — Mt rps13 distribution among rosids. The figure shows losses of mt rps13 among rosids in a phylogenetic context, and the inferred timing of numit rps13 formation by gene duplication. [file 1471-2148-8-25-S1.PDF]

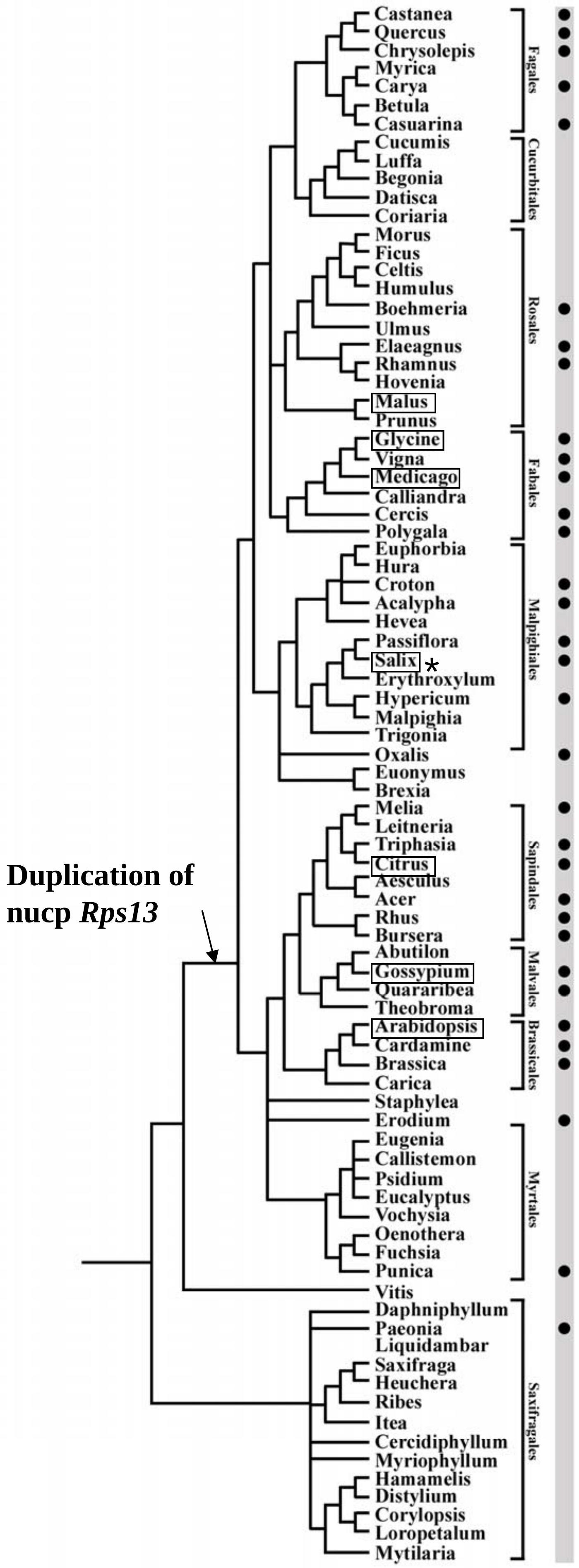


**Additional file 1:**
**Phylogenetic tree showing losses of mt *rps13* among rosids.** Bullets indicate loss of mt *rps13* as judged by Southern blot hybridizations. Data are from Adams et al. (2002) and this figure was modified and redrawn from a figure in that paper. Rosid species included in this study are shown by rectangular boxes. Note that *Populus* is closely related to *Salix* (Asterisk). Reference: Adams K, Qiu Y, Stoutemyer M, Palmer J: Punctuated evolution of mitochondrial gene content: high and variable rates of mitochondrial gene Loss and transfer to the nucleus during angiosperm evolution. *Proc Natl Acad Sci USA* 2002, 99:9905-9912.
