# Supplementary material for: Molecular adaptation and expression evolution following duplication of genes for organellar ribosomal protein S13 in rosids
Source: BMC Evol Biol. 2008 Jan 26;8:25. doi: 10.1186/1471-2148-8-25 (PMC2258280; doi:10.1186/1471-2148-8-25)
Supplement: Additional file 2 — Pairwise Ka and Ks analyses. The table shows the results of Ka analysis and Ks analysis for numit rps13 and nucp rps13 in seven rosid species. [file 1471-2148-8-25-S2.PDF]

**Additional file 2: Comparison of $K_a$ and $K_s$ values between nucp *Rps13* and numt *Rps13*.**

| Variable | Taxon | *Arabidopsis* | *Citrus* | *Glycine* | *Gossypium* | *Malus* | *Medicago* | *Populus* |
|---|---|---|---|---|---|---|---|---|
| | | | | nucp *rps13* (0.10 ± 0.03)[a] | | | | |
| | *Arabidopsis* | - | | | | | | |
| | *Citrus* | 0.0656 | - | | | | | |
| | *Glycine* | 0.1045 | 0.1144 | - | | | | |
| | *Gossypium* | 0.0939 | 0.0472 | 0.1042 | - | | | |
| | *Malus* | 0.1245 | 0.0898 | 0.1333 | 0.0715 | - | | |
| | *Medicago* | 0.1336 | 0.1398 | 0.071 | 0.1327 | 0.1317 | - | |
| | *Populus* | 0.1084 | 0.085 | 0.1256 | 0.0758 | 0.0917 | 0.1247 | - |
| $K_a$ | | | | numt *rps13* (0.40 ± 0.10) | | | | |
| | *Arabidopsis* | - | | | | | | |
| | *Citrus* | 0.3220 | - | | | | | |
| | *Glycine* | 0.5687 | 0.4275 | - | | | | |
| | *Gossypium* | 0.4534 | 0.3343 | 0.459 | - | | | |
| | *Malus* | 0.5146 | 0.4485 | 0.4149 | 0.4801 | - | | |
| | *Medicago* | 0.4875 | 0.4019 | 0.0858 | 0.4320 | 0.3893 | - | |
| | *Populus* | 0.4071 | 0.2355 | 0.4462 | 0.3677 | 0.4187 | 0.3871 | - |
| | | | | nucp *rps13* (1.90 ± 0.81) | | | | |
| | *Arabidopsis* | - | | | | | | |
| | *Citrus* | 3.3196 | - | | | | | |
| | *Glycine* | 3.3215 | 1.0258 | - | | | | |
| | *Gossypium* | 2.0613 | 0.9158 | 1.3618 | - | | | |
| | *Malus* | 3.3534 | 1.8194 | 1.6386 | 1.1956 | - | | |
| | *Medicago* | 3.2608 | 1.6585 | 1.1834 | 2.2887 | 1.5414 | - | |
| | *Populus* | 2.2241 | 0.7589 | 2.022 | 1.5312 | 1.4986 | 1.8353 | - |
| $K_s$ | | | | numt *rps13* (1.57 ± 0.45) | | | | |
| | *Arabidopsis* | - | | | | | | |
| | *Citrus* | 1.1649 | - | | | | | |
| | *Glycine* | 1.7145 | 1.6867 | - | | | | |
| | *Gossypium* | 1.7634 | 1.0827 | 1.5318 | - | | | |
| | *Malus* | 1.7192 | 1.4132 | 1.7408 | 1.0253 | - | | |
| | *Medicago* | 1.8233 | 2.2337 | 2.6241 | 1.2828 | 1.4212 | - | |
| | *Populus* | 1.0634 | 0.9023 | 1.8488 | 1.2482 | 1.3868 | 2.2735 | - |

[a]The mean and standard deviation are shown in parentheses.
