# Supplementary material for: Molecular adaptation and expression evolution following duplication of genes for organellar ribosomal protein S13 in rosids
Source: BMC Evol Biol. 2008 Jan 26;8:25. doi: 10.1186/1471-2148-8-25 (PMC2258280; doi:10.1186/1471-2148-8-25)
Supplement: Additional file 3 — Microarray data legend. The table lists the organs and developmental stages from Arabidopsis thaliana for which microarray data were analyzed. [file 1471-2148-8-25-S3.PDF]

Additional file 3: Microarray data legend.

O1: cotyledons (7d)
O2: hypocotyls (7d)
O3: roots (7d)
O4: vegetative leaves on shoot apex (7d)
O5: leaves 1-2
O6: vegetative shoots on shoot apex (7d)
O7: seedling green shoots (7d)
O8: shoot apexes before bolting (14d)
O9: roots (17d)
O10: rosette #4 young leaves (10d)
O11: rosette #2 mature leaves (17d)
O12: rosette #4 mature leaves (17d)
O13: rosette #6 mature leaves (17d)
O14: rosette #8 young leaves (17d)
O15: rosette #10 young leaves (17d)
O16: rosette #12 young leaves (17d)
O17: petioles on leaf #7 (17d)
O18: proximal half young leaves on leaf #7 (17d)
O19: distal half young leaves on leaf #7 (17d)
O20: senescence leaves (35d)
O21: cauline leaves (21+d)
O22: stems on the second internode shoot (21+d)
O23: stems on the first internode shoot (21+d)
O24: shoot apexes after bolting (21d)
O25: stage 9 flowers (21+d)
O26: stage 10-11 flowers (21+d)
O27: stage 12 flowers (21+d)
O28: sepals on stage 12 flower (21+d)
O29: petals on stage 12 flower (21+d)
O30: stamens on stage 12 flower (21+d)
O31: carpals on stage 12 flower (21+d)
O32: stage 15 flowers (21+d)
O33: pedicles on stage 15 flower (21+d)
O34: sepals on stage 15 flower (21+d)
O35: pedals on stage 15 flower (21+d)
O36: stamens on stage 15 flower (21+d)
O37: carpals on stage 15 flower (21+d)
O38: pollens (6 wk)
O39: globular embryos on stage 3 silique (8wk)
O40: heart embryos on stage 4 silique (8wk)
O41: triangle embryos on stage 5 silique (8wk)
O42: torpedo embryos on stage 6 seed (8 wk)
O43: walking stick seeds on stage 7 seed (8wk)
O44: early curl codyledons on stage 8 seed (8wk)
O45: early green codyledons on stage 9 seed (8wk)
O46: green codyledons on stage 10 seed (8wk)
O47: early rosettes (7d)
O48: middle rosettes (14d)
O49: late rosettes (21d)
O50: leaves (15d)
O51: stage 12 flowers (4wk)
