# Supplementary material for: Molecular adaptation and expression evolution following duplication of genes for organellar ribosomal protein S13 in rosids
Source: BMC Evol Biol. 2008 Jan 26;8:25. doi: 10.1186/1471-2148-8-25 (PMC2258280; doi:10.1186/1471-2148-8-25)
Supplement: Additional file 4 — RNA editing of mt rps13 in Malus compared with other flowering plants. The figure shows an alignment of mt rps13 from several land plants, indicating the positions of RNA editing sites in Malus, and a list of mt rps13 RNA editing sites from several angiosperms. [file 1471-2148-8-25-S4.PDF]

(A)

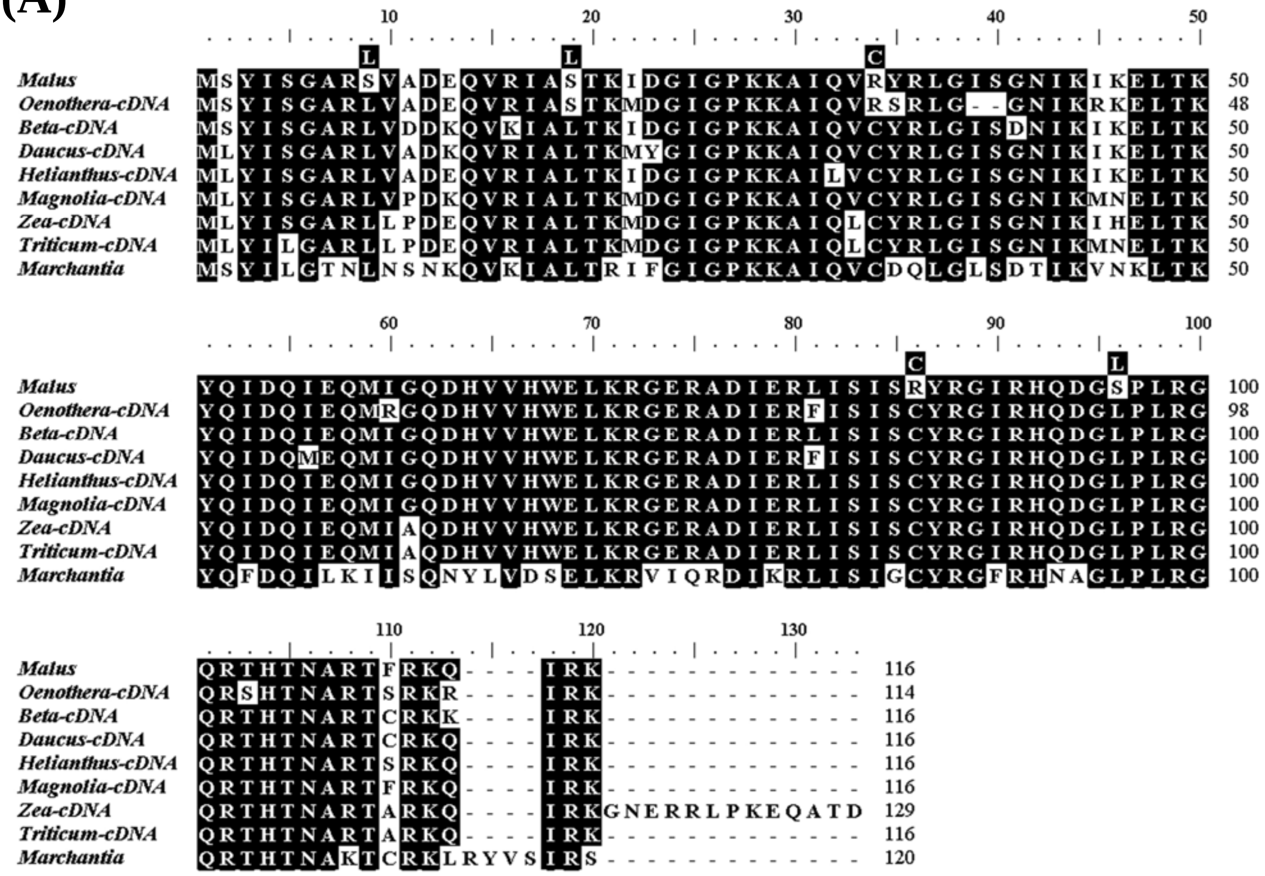

(B)

RNA editing of mt *rps13* among different plant species.

Codon	*Malus*	*Oenothera*	*Beta*	*Daucus*	*Helianthus*	*Magnolia*	*Triticum*	*Zea*
2	TCA (S)	TCA (S)	TCA (S)	TTA (L)	TTA (L)	TCA (S→L)	TCA (S→L)	TCA (S→L)
5	TCA (S)	TCA (S)	TCA (S)	TCA (S)	TCA (S)	TCA (S)	TCA (S→L)	TCA (S)
9	TCA (S→L)	TCA (S→L)	TTA (L)	TCA (S→L)	TCA (S→L)	TCA (S→L)	TCA (S→L)	TCA (S→L)
19	TCA (S→L)	TCA (S)	TCA (S→L)	TCA (S→L)	TCA (S→L)	TCA (S→L)	TCA (S→L)	TCA (S→L)
34	CGT (R→C)	CGT (R)	TGT (C)	CGT (R→C)	CGT (R→C)	CGT (R→C)	CGT (R→C)	CGT (R→C)
78	ATC (I)	ATC (I)	ATC (I→I)	ATC (I)	ATC (I)	ATC (I)	ATC (I)	ATC (I)
81	TTA (L)	TTC (F→F)	TTA (L)	TTC (F→F)	TTA (L)	TTA (L)	TTA (L)	TTA (L)
86	CGT (R→C)	TGT (C)	TGT (C)	TGT (C)	TGT (C)	CGT (R→C)	CGT (R→C)	CGT (R→C)
96	TCG (S→L)	TCG (S→L)	TTG (L)	TTG (L)	TCA (S→L)	TCG (S→L)	TCG (S→L)	TCG (S→L)

Additional file 4:
RNA editing of mitochondrial *rps13* in *Malus domestica* compared with other plants. A) RPS13 sequences of *Malus domestica* deduced from genomic DNA sequence are aligned with deduced cDNA sequences of seven rosid species and the genomic sequence of *Marchantia polymorpha*. Amino acids changed by RNA editing for *Malus domestica* are given above the genomic DNA sequence. Identical amino acids are shaded in black. Dots refer to gaps inserted to improve the alignment or missing amino acids. Numbers of amino acids are indicated on the right side of each sequence. B) Genomic triplets are shown for an RNA editing site in at least one plant species. Underlined nucleotide indicates the site where a C-to-U transition occurs. The corresponding amino acid change is indicated and signified by the single letter code in parenthesis.
