# Supplementary figures and images for: Molecular adaptation and expression evolution following duplication of genes for organellar ribosomal protein S13 in rosids
Source: BMC Evol Biol. 2008 Jan 26;8:25. doi: 10.1186/1471-2148-8-25 (PMC2258280; doi:10.1186/1471-2148-8-25)

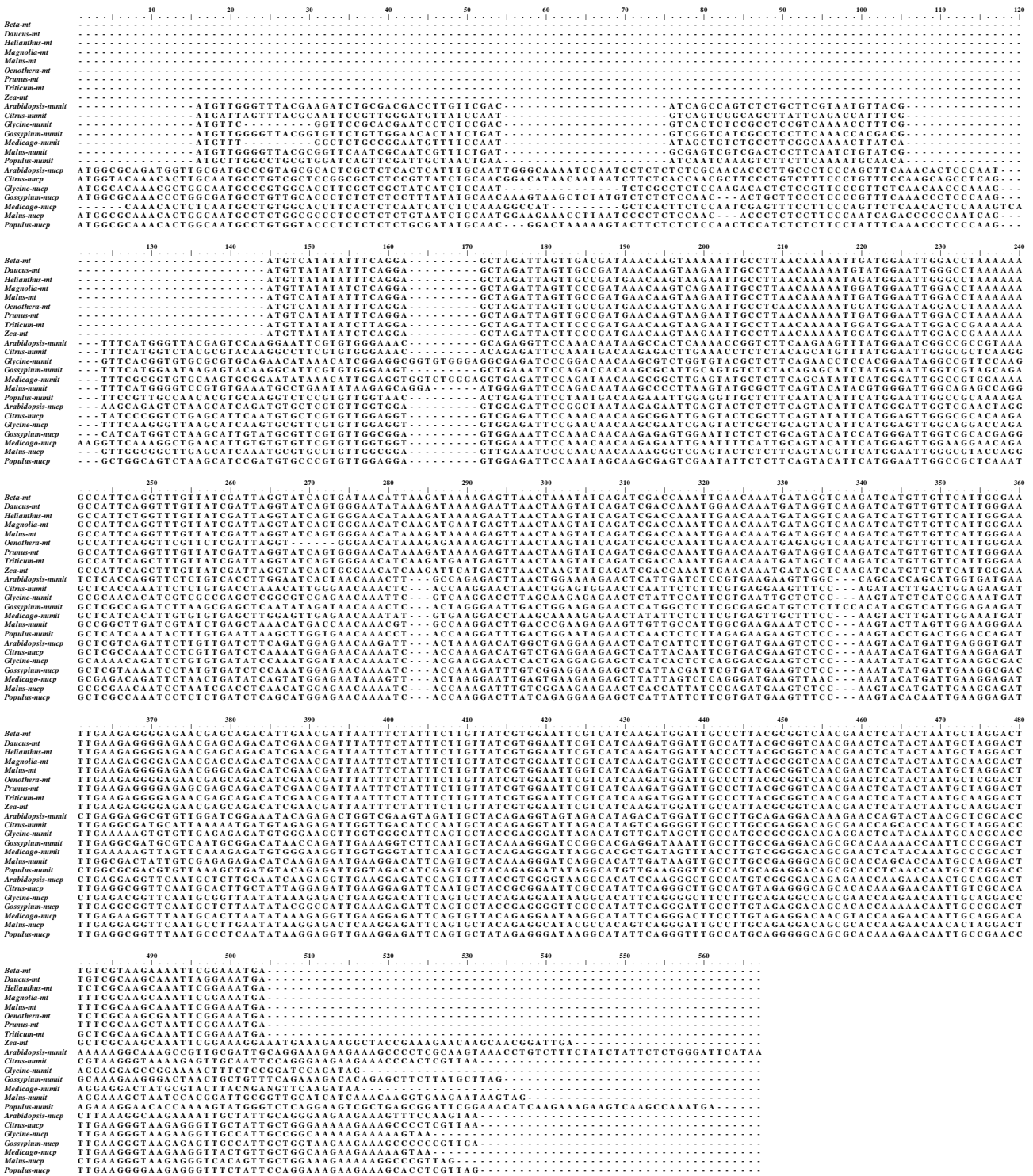

Additional file 5:  
Sequence alignment.

Supplement: Additional file 5 — Sequence alignment. The figure shows an alignment of numit rps13, nucp rps13, and cDNAs of mt rps13 for taxa analyzed in this study. The targeting sequences at the 5' end of the numit rps13 and nucp rps13 genes do not align with each other and were excluded from Ka/Ks analysis. [file 1471-2148-8-25-S5.PDF]
