# Supplementary material for: Molecular adaptation and expression evolution following duplication of genes for organellar ribosomal protein S13 in rosids
Source: BMC Evol Biol. 2008 Jan 26;8:25. doi: 10.1186/1471-2148-8-25 (PMC2258280; doi:10.1186/1471-2148-8-25)
Supplement: Additional file 6 — RT-PCR primers. The table lists the primers used for RT-PCR and qRT-PCR experiments. [file 1471-2148-8-25-S6.PDF]

Additional file 6: RT-PCR primers.

Primer ID	Gene	Sequence	Orientation
RT-PCR			
MalusRps13NuF1	numt *rps13*	5'- GTTGGGGTTACGCGGTTCAATCG -3'	Forward
MalusRps13NuF2	numt *rps13*	5'- CACGAGGGGCAAAATCTAAGTATCCA -3'	Forward
MalusRps13NuR1	numt *rps13*	5'- AATAAGTGGTAAGACATCTAACAACC -3'	Reverse
MalusRps13NuR2	numt *rps13*	5'- CACCTTGTTTGATGATGCAACCGCAATC -3'	Reverse
MalusRps13MtF1	mt *rps13*	5'-GATCATCAGAGAGGAGACAG-3'	Forward
MalusRps13MtF2	mt *rps13*	5'-TCAGGAGCTAGATCAGTTGCCGA-3'	Forward
MalusRps13MtR1	mt *rps13*	5'-TTAGTATGAGTTCGTTGACCG-3'	Reverse
MalusRps13MtR2	mt *rps13*	5'-GAAGCTATCAGTGTGATTCATCAGAC-3'	Reverse
qRT-PCR			
MalusACT2F1	*ACT2*	5'-AATGGTGAAGGCTGGATTTGCTGG-3'	Forward
MalusACT2R1	*ACT2*	5'- TGACCCATACCAACCATGACACCA-3'	Reverse
MalusRps10F1	numt *rps10*	5'- CAAGGAAGATTGCACTGCCGGAAT-3'	Forward
MalusRps10R1	numt *rps10*	5'- CGTATTGGGCTCCAAATATGCGCT-3'	Reverse
qNumtRps13F1	numt *rps13*	5'- CGCGGTTCAATCGCAATCGTTTCT-3'	Forward
qNumtRps13R1	numt *rps13*	5'-ATCCCACGTATGTACTGAAGCGC-3'	Reverse
qMtRps13F1	mt *rps13*	5'- AGGAGCTAGATCAGTTGCCGATGA-3'	Forward
qMtRps13R1	mt *rps13*	5'- CCTAATCGATAACGAACCTGAATGGC-3'	Reverse
